# Changes in Pediatric Acute Respiratory Infections Before, During, and After the COVID-19 Pandemic: A Retrospective Study

**DOI:** 10.7759/cureus.92841

**Published:** 2025-09-21

**Authors:** Ana Fradique, Marta Rodrigues, Laura Isabel Azevedo e Sousa, Arminda Jorge

**Affiliations:** 1 Pediatrics, Unidade Local de Saúde de Coimbra, Coimbra, PRT; 2 Pediatrics, Unidade Local de Saúde da Cova da Beira, Covilhã, PRT; 3 Health Sciences, Universidade da Beira Interior, Covilhã, PRT; 4 Pediatrics, Centro Hospitalar e Universitário Cova da Beira, Covilhã, PRT

**Keywords:** covid-19, epidemiology, non-pharmacological interventions, pediatrics, respiratory infections

## Abstract

Introduction and objectives: Acute respiratory infections (ARIs) are a significant cause of morbidity in children and a common reason for emergency department (ED) visits. During the COVID-19 pandemic, governments adopted non-pharmacological interventions (NPIs) to limit SARS-CoV-2 transmission. These measures also reduced the circulation and seasonality of other respiratory pathogens, with little impact on infections in non-respiratory organ systems. This study aimed to quantify changes in the incidence and severity of pediatric ARIs before, during, and after the pandemic.

Materials and methods: We conducted a retrospective observational time-series study of children aged 0-10 years presenting with ARIs to a level 2 pediatric ED in Covilhã, Portugal, from April 2019 to April 2023. To ensure representativeness while controlling data volume, admissions from the first five days of each month were analyzed. Three periods were defined: pre-COVID-19 (Pre-C), COVID-19 (C), and post-COVID-19 (Post-C). All statistical analyses were performed using SPSS Statistics version 28 (IBM Corp. Released 2021. IBM SPSS Statistics for Windows, Version 28.0. Armonk, NY: IBM Corp.). A significance level of p = 0.05 was applied.

Results: Among 7,448 sampled ED visits, 2,210 (29.7%) involved ARIs. Their proportion fell from Pre-C to C (p = 0.028) and rose sharply in Post-C (p < 0.001), exceeding Pre-C levels (p < 0.001). In C, atypical inter-seasonal peaks occurred. Hospitalization rates, length of stay, and oxygen requirements showed no significant variation.

Conclusions: COVID-19 NPIs were associated with a marked reduction and altered timing of pediatric ARIs, whereas infections in other organ systems were largely unaffected. After restrictions were lifted, ARI incidence surpassed pre-pandemic levels, but disease severity remained stable. These findings highlight how public health measures and subsequent exposure shifts can reshape respiratory infection epidemiology in children.

## Introduction

Acute respiratory infections (ARIs) are among the most common illnesses worldwide and a leading cause of pediatric morbidity and mortality, accounting for an estimated 1.9 million deaths in children under five globally each year and a substantial burden on health services in Europe [1,2]. Their incidence is highest in early childhood due to immature immunity and narrow airways, gradually declining with age [3,4]. ARIs may involve the upper or lower respiratory tract: upper infections (e.g., rhinorrhea, sore throat, cough) are usually self-limited, whereas lower infections can progress to severe disease, including acute respiratory distress syndrome [5]. Most cases are viral, although mixed infections and bacterial complications are frequent [1,6].

In late 2019, SARS-CoV-2 emerged, resulting in the COVID-19 pandemic. On February 11, 2020, the World Health Organization declared it a pandemic [7,8]. To curb transmission, governments adopted non-pharmacological interventions (NPIs), such as physical distancing, mask use, hand hygiene, and school or activity closures [9]. In Portugal, a state of emergency was declared on March 18, 2020, followed by the implementation of progressive mask mandates [10-12].

While NPIs substantially reduced SARS-CoV-2 spread, they also disrupted the circulation of other respiratory pathogens in children [13,14], creating an “immunity gap” [15]. After restrictions were eased, ARIs re-emerged, often outside their usual seasonal peaks, with incidence exceeding pre-pandemic levels [16,17].

To better understand these trends, we analyzed ARIs in children aged 0-10 years attending a level 2 pediatric emergency department (ED) across three distinct periods: Pre-COVID-19 (Pre-C), spanning from April 2019 to March 2020, representing the 12 months before the implementation of COVID-19 containment measures in Portugal [18]; COVID-19 (C), from April 2020 to April 2022, 25 months marked by multiple pandemic waves during which the government implemented and gradually eased restrictions such as lockdowns, travel bans, social distancing, and mandatory mask use in public spaces; and Post-COVID-19 (Post-C), from May 2022 to April 2023, the 12 months following the removal of most restrictions, including the government’s decision on April 21, 2022, to end the mandatory use of masks indoors [19].

The primary objective of this study was to assess changes in the frequency and pattern of pediatric respiratory infectious diseases across these periods. Secondary objectives included evaluating differences in hospitalization rates, length of stay, and the distribution of specific respiratory pathogens.

## Materials and methods

Study design and setting

This was a retrospective observational study conducted at the Centro Hospitalar Cova da Beira, a level 2 hospital located in Covilhã, Portugal, and the study period extended from April 1, 2019, to April 30, 2023, encompassing the pre-pandemic, pandemic, and post-pandemic phases of COVID-19.

Participants

The study population included all pediatric patients under the age of 10 years who were admitted to the hospital with a primary diagnosis of respiratory infectious disease during the study period. Patients aged 10 years or older and those with incomplete medical records were excluded from the analysis.

Data sources and variables

Clinical data were retrieved from the hospital’s electronic medical record (SClínico Hospitalar®, Portugal). To balance representativeness and feasibility, we sampled the first five days of each month. This approach ensured even temporal coverage, reduced the risk of missing documentation due to high-volume days, and aligned with staffing patterns that guaranteed consistent triage and coding practices. Variables recorded included demographic characteristics (age, sex), admission and discharge dates, primary and secondary diagnoses, laboratory and microbiological findings, treatments received, length of hospital stay, and clinical outcomes. Respiratory infectious diseases were classified according to the International Classification of Diseases (ICD-10) diagnostic codes recorded at discharge.

Study period definitions

For analytical purposes, the study period was divided into three phases: (1) pre-COVID-19 (Pre-C), from April 2019 to March 2020, representing the 12 months before COVID-19 containment measures were first implemented in Portugal [18]; (2) COVID-19 (C), from April 2020 to April 2022, a 25-month period during which Portugal experienced multiple COVID-19 waves. During this time, the government adopted a phased approach to imposing and easing restrictions, including lockdowns, travel bans, social distancing measures, and mandatory mask use in public spaces; and (3) post-COVID-19 (Post-C), from May 2022 to April 2023, covering the 12 months following the removal of most pandemic-related restrictions. On April 21, 2022, the government ended the mandatory use of masks indoors [19].

Statistical analysis

Data analysis was performed using SPSS Statistics version 28 (IBM Corp. Released 2021. IBM SPSS Statistics for Windows, Version 28.0. Armonk, NY: IBM Corp.). Categorical variables were summarized as frequencies and percentages, while continuous variables were expressed as medians with interquartile ranges (IQR) or means with standard deviations (SD), as appropriate. Comparisons between groups were performed using the chi-square test or Fisher’s exact test for categorical variables and the Mann-Whitney U test or independent samples t-test for continuous variables, depending on data distribution. A p-value < 0.05 was considered statistically significant.

Ethics approval

The study was conducted in accordance with the ethical standards outlined in the Declaration of Helsinki. It was approved by the Ethics Committee of the Centro Hospitalar Universitário Cova da Beira (opinion no. 41/2023, dated July 19, 2023). As this was a retrospective study using anonymized data, the requirement for informed consent was waived.

## Results

Using the four-year study period, 7,448 pediatric ED visits were analyzed (first five days of each month). Of these, 2,210 (29.7%) involved ARIs. In this study, ED visits refer to all pediatric presentations recorded during the sampled days. In contrast, ARI-related visits denote those in which an ARI was diagnosed, regardless of subsequent hospitalization. Among these ARI-related visits, 57 children (2.6%) required admission to the pediatric ward; the remaining cases were discharged from the ED.

Of the 2,210 ARI-related visits, 1,014 (45.9%) were female and 1,196 (54.1%) were male. Ages ranged from 0 to 10 years (mean 3.61, SD 2.87), with 425 (19.2%) individuals under one year of age. No significant differences were observed in the age distribution across the three periods (p = 0.051).

**Figure 1:**
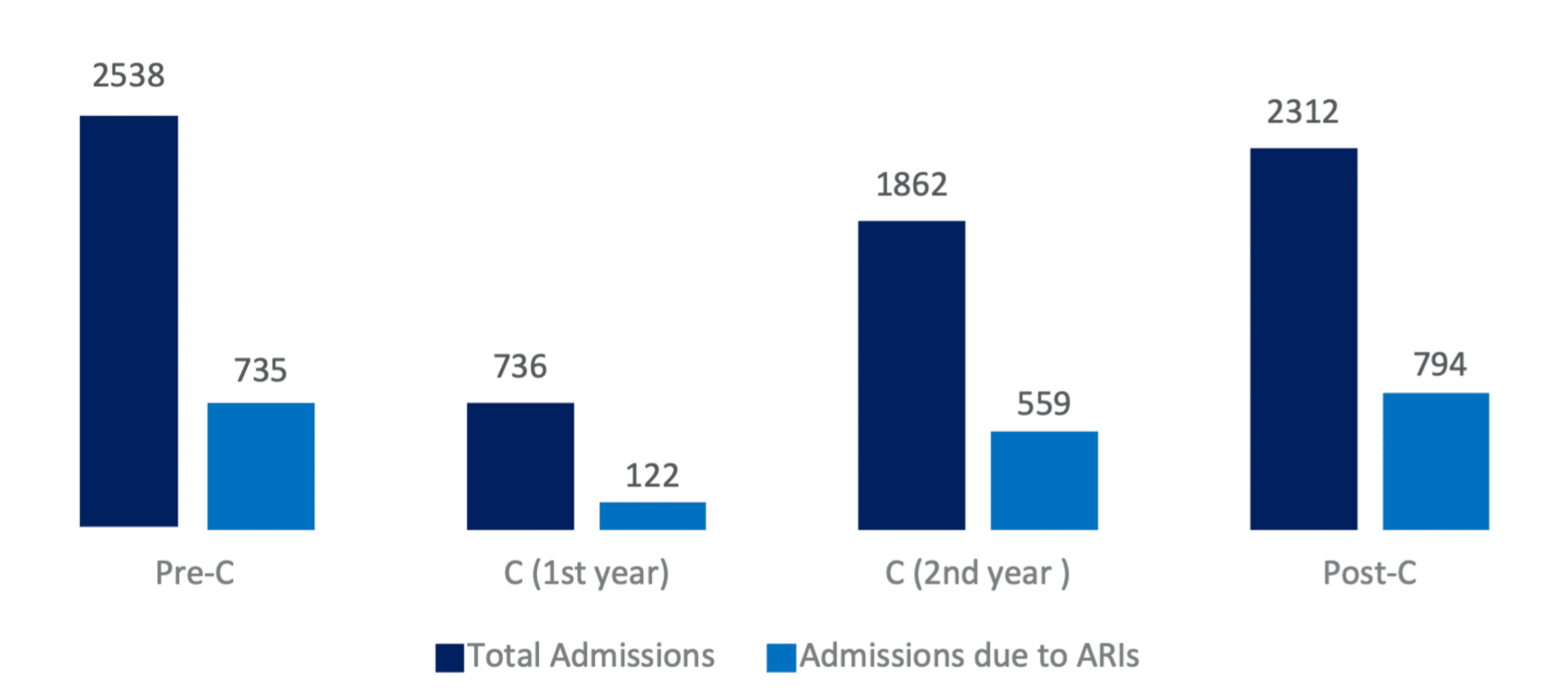
Total number of admissions and number of ARI-related admissions in the ED Figure 1 illustrates the total number of ED admissions (dark blue bars) and the number of admissions due to ARIs (light blue bars) during the three study periods: Pre-C, C (first and second years shown separately), and Post-C. This figure provides a visual comparison of the burden of ARI cases relative to all ED admissions over time. --> Error bars (or values in brackets) indicate the 95% confidence intervals for the proportion of ARIs: Pre-C 29.0% (27.2–30.7), C (1st year) 16.6% (13.9–19.3), C (2nd year) 30.0% (27.9–32.1), and Post-C 34.3% (32.4–36.3). ARI: acute respiratory infection, ED: emergency department, Pre-C: pre-COVID-19, C: COVID-19, Post-C: post-COVID-19

Figure 1 shows the variation in the total number of ED admissions, as well as ARI-related admissions, over the three time periods. ARI-related admissions corresponded to 29.0%, 26.2%, and 34.3% of the total number of ED admissions in Pre-C, C, and Post-C, respectively. In C, two phases were considered: a first confinement phase, from April 2020 to March 2021, with 16.6% of ARI-related admissions, and a second phase, from April 2021 to April 2022, with 30.0%. The daily average number of children attending the ED for ARIs was 12, 2, 8, and 13 for Pre-C, C (phase 1), C (phase 2), and Post-C, respectively.

A significant difference was observed in the incidence of ARI-related admissions relative to the total number of ED admissions among the three time periods. The proportion of ARIs significantly decreased from Pre-C to C (p = 0.028), followed by a significant increase from C to Post-C (p < 0.001); similarly, the comparison between Pre-C and Post-C shows a significant increase in the proportion of ARIs (p < 0.001), reflecting a trend of increased ARI incidence after the pandemic.

**Figure 2:**
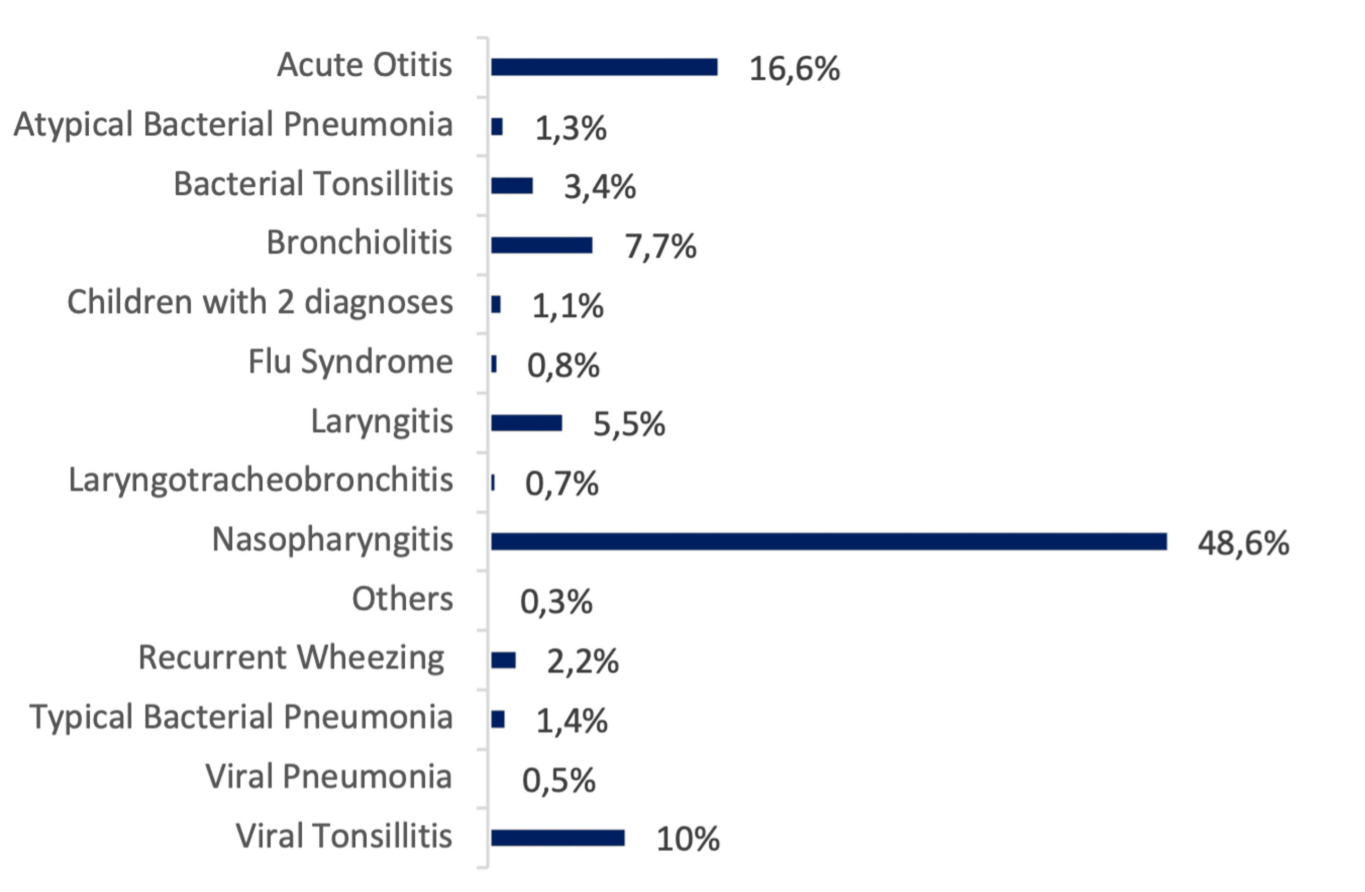
Percentage distribution of ARIs that prompted visits to the ED Figure 2 shows the proportional distribution of ARI diagnoses across the study population. Each bar represents the percentage of children with a given ARI diagnosis relative to the total ARI cases. The figure highlights the predominance of nasopharyngitis, followed by acute otitis, tonsillitis, bronchiolitis, and laryngitis. Less frequent conditions, including atypical bacterial pneumonia and viral pneumonia, are also displayed. This figure allows for a direct appreciation of the relative frequency of each diagnosis. ARI: acute respiratory infection, ED: emergency department

Figure 2 presents a detailed distribution of ARIs leading to admission. The most reported pathologies were nasopharyngitis (48.6%, n = 1,075), acute otitis (16.6%, n = 366), and viral tonsillitis (10%, n = 221).

The distribution of emergency episodes across the three periods (Table 1) revealed a decrease in cases from Pre-C to C for most pathologies, except for recurrent wheezing, laryngotracheobronchitis, nasopharyngitis, typical bacterial pneumonia, and the category of children with two diagnoses. Comparing C and Post-C, there was an increase in ARI incidence, except for viral tonsillitis, laryngotracheobronchitis, atypical bacterial pneumonia, flu-like syndrome, and the category of others. There was also an increase in incidence between Pre-C and Post-C in episodes of bacterial tonsillitis, recurrent wheezing, nasopharyngitis, viral and typical bacterial pneumonia, and the category of children with two diagnoses.

**Table 1:** Distribution of the number/percentage of ARIs across the three study periods Table 1 presents the absolute number and percentage of ARIs diagnosed in the ED during the three study periods (Pre-C, C, and Post-C). Diagnoses were classified into specific clinical categories, and their relative frequencies are shown for each period and overall. The table allows comparison of ARI patterns Pre-C, C, and Post-C. Percentages were calculated relative to the total ARI cases in each period. ARI: acute respiratory infection, ED: emergency department, Pre-C: pre-COVID-19, C: COVID-19, Post-C: post-COVID-19

			Pre-C	C	Post-C	Total
			n (%)	n (%)	n (%)	n
Acute otitis			133 (36.3%)	116 (31.7%)	117 (32.0%)	366
Bronchiolitis			73 (42.7%)	36 (21%)	62 (36.3%)	171
Children with two diagnoses			4 (16%)	7 (28%)	14 (56%)	25
Flu syndrome			10 (55.6%)	4 (22.2%)	4 (22.2%)	18
Laryngitis			57 (46.7%)	29 (23.8%)	36 (29.5%)	122
Laryngotracheobronchitis			6 (40%)	7 (46.7%)	2 (13.3%)	15
Nasopharyngitis			291 (27.1%)	367 (34.1%)	417 (38.8%)	1075
Others			2 (66.7%)	1 (33.3%)	0 (0%)	3
Pneumonia	Viral		2 (20%)	1 (10%)	7 (70%)	10
	Bacterial	Typical	10 (32.3%)	10 (32.3%)	11 (35.5%)	31
		Atypical	13 (44.8%)	9 (31%)	7 (24.1 %)	29
Recurrent wheezing			12 (24.5%)	18 (36.7%)	19 (38.8%)	49
Tonsillitis	Viral		89 (40.3%)	71 (32.1%)	61 (27.6%)	221
	Bacterial		33 (44%)	5 (6.7%)	37 (49.3%)	75
Total			735	681	794	2210

Despite the fluctuations mentioned, few pathologies showed statistically significant changes between time periods in the proportion of cases relative to the total number of admissions. Table 2 shows a significant decrease in bacterial tonsillitis and acute bronchiolitis during the pandemic, followed by a significant increase during Post-C. Additionally, laryngitis showed a significant decrease from Pre-C to C, while viral pneumonia showed a significant increase from C to Post-C. The diagnosis of nasopharyngitis stands out, revealing a significant increase across periods.

**Table 2:** Statistical analysis of ARI incidence variation (as a proportion of total ED admissions) between the different time periods Table 2 compares the incidence of each ARI diagnosis relative to the total number of ED admissions across three periods (Pre-C, C, and Post-C). Statistical comparisons between periods were performed using the chi-square test. For categories with expected frequencies <5, Fisher’s exact test was applied. The test statistic (χ² value or Fisher’s exact) and corresponding p-values are reported for each pairwise comparison. This table highlights which ARI diagnoses showed significant variation in incidence across time. ARI: acute respiratory infection, ED: emergency department, Pre-C: pre-COVID-19, C: COVID-19, Post-C: post-COVID-19

		Pre-C vs. C	C vs. Post-C	Pre-C vs. Post-C
Diagnosis	χ²	p-value	p-value	p-value
Acute otitis	3.28	0.092	0.134	0.880
Bronchiolitis	10.70	<0.001	0.001	0.681
Children with two diagnoses	Fisher's exact test	0.386	0.072	0.010
Flu syndrome	Fisher's exact test	0.142	0.079	0.827
Laryngitis	10.60	0.002	0.177	0.081
Laryngotracheobronchitis	Fisher's exact test	0.049	0.079	0.861
Nasopharyngitis	36.39	0.004	<0.001	<0.001
Others	Fisher's exact test	0.609	-	0.141
Viral pneumonia	Fisher's exact test	0.300	0.027	0.235
Bacterial pneumonia typical	0.03	0.609	0.120	0.055
Bacterial pneumonia atypical	2.32	0.497	0.127	0.416
Recurrent wheezing	9.96	0.301	0.602	0.128
Tonsillitis viral	8.51	0.111	0.838	0.081
Tonsillitis bacterial	21.27	<0.001	<0.001	0.381

Throughout the three periods, viral research in respiratory secretions and rapid antigen diagnostic testing in the oropharynx were conducted to detect the etiological agents behind ARIs. Out of 452 children tested, 169 (37.4%) tested positive for viruses (19 of which for more than one virus) and 77 (17%) for bacteria. Regarding the causative agents of viral ARIs, 12 different viruses were identified, with the most common being respiratory syncytial virus (RSV) (n = 67), SARS-CoV-2 (n = 44), adenovirus (n = 20), and influenza A (n = 19). Testing for respiratory pathogens was performed at the discretion of the attending physician, usually in children with moderate/severe symptoms, a need for hospitalization, or an unclear diagnosis. Therefore, pathogen distribution reflects a clinically selected subset and may not represent the entire ARI cohort. Among the bacterial agents, 76 were identified as *Streptococcus pyogenes* in the oropharynx. Pathogenic agents were predominantly identified in children with conditions such as nasopharyngitis, tonsillitis, and bronchiolitis. SARS-CoV-2 and RSV viruses stood out as the leading causes of nasopharyngitis. Among the viruses causing bronchiolitis, RSV had the most significant impact.

The number of microbial agents identified successively increased over time: Pre-C (n = 64), C (n = 94), and Post-C (n = 113). In part, the emergence of the new SARS-CoV-2 virus was responsible for the increase from Pre-C to C; however, in C, RSV was the most representative agent. In Post-C, the number of SARS-CoV-2 infections reduced by half.

There were 57 (2.6%) hospitalizations throughout the period studied. On average, children stayed in the hospital for 3.82 days (range: 1-15 days). During Pre-C, there were 24 hospitalizations (42.1%), with three complications reported. There were 16 (28.1%) and 17 (29.8%) hospitalizations during C and Post-C. ARI-related hospitalizations corresponded to 0.95%, 0.62%, and 0.74% of the total number of admissions during Pre-C, C, and Post-C, respectively. When comparing the total number of hospitalizations between the three periods, no statistically significant changes were observed (χ² = 2.13, df = 2, p = 0.345). The length of hospital stays also remained constant (one-way ANOVA, p = 0.565). Similarly, the need for oxygen therapy showed no significant differences across periods (χ² = 3.79, df = 2, p = 0.150). Complications were only reported during the Pre-C period (n = 3).

**Figure 3:**
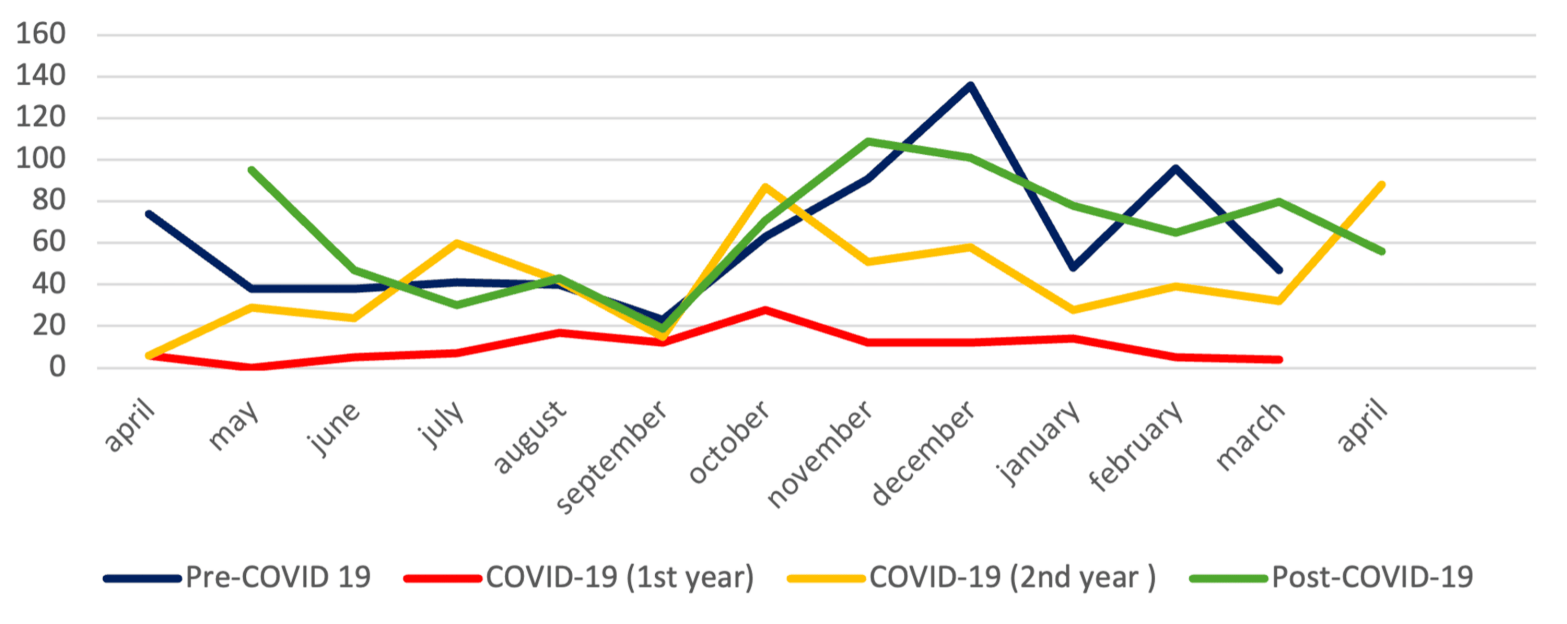
Incidence of ARIs over time Figure 3 presents the monthly distribution of ARI cases across the study periods (Pre-C, C first year, C second year, and Post-C). Each colored line represents one period, allowing comparison of seasonal variation in ARI incidence Pre-C, C, and Post-C. Peaks and troughs across months highlight the shift in epidemic curves, with an atypical decrease in C and resumption of seasonal peaks in Post-C. ARI: acute respiratory infection, Pre-C: pre-COVID-19, C: C, Post-C: post-COVID-19

A variation in the incidence of ARIs over the four years can be observed (Figure 3). In Pre-C, there was a high number of cases (n = 74) in early spring (April) and a decrease in cases over the next five months, with a re-increase in October (n = 63), November (n = 91), December (n = 136), and the following February (n = 96). The peak incidence of Pre-C occurred in December. In C, during both years, the peak incidence was in October (28 cases in 2020 and 87 cases in 2021), with a subsequent case decrease in the colder months. During this period, the usual increase in cases at the beginning of the spring was not observed. In the first year of C, there was an increase in cases in August (n = 17), and, in the second year, there was an increase in July (n = 60). At the end of C (April 2022) and the beginning of Post-C (May 2022), there was an increase in ARIs, with a peak in May (n = 95), followed by a decrease. In October 2022, the number of episodes started to increase, with the peak incidence in November (n = 109), followed by a high number of episodes in the colder months and a new increase in cases in March (n = 80).

## Discussion

Based on the data obtained, a sharp decrease in the number of ARI-related visits to the ED was observed during the first year of COVID-19. This decline likely reflects the impact of strict containment measures on children’s daily routines and on the adults who care for them. School closures, teleworking, limits on public circulation, and suspension of group activities reduced opportunities for pathogen transmission [18]. In addition, changes in healthcare-seeking behavior, such as increased use of telehealth, calls to the NHS helpline, and parental reluctance to visit crowded EDs for mild symptoms, may also have contributed to the fall in attendances [19].

A marked 4.6-fold rise in ARI visits between the first and second years of the pandemic coincided with the progressive relaxation of restrictions and the gradual return to in-person schooling. Nevertheless, when the entire 25-month pandemic was compared with the 12-month pre-pandemic period, a significant overall decline in ARIs remained, echoing findings from other national and international studies [20-23].

In Post-C, after most restrictions were lifted, ARI incidence increased significantly. When compared with Pre-C, Post-C showed higher proportions of ARI-related visits relative to total ED visits. Despite this resurgence, there was no evidence of greater disease severity: hospitalization rates, length of stay, oxygen requirement, and complications were similar to pre-pandemic values. Only nasopharyngitis and the category of children with two diagnoses increased significantly, both of which are generally mild conditions. These findings align with a German study reporting a rise in upper respiratory tract infections between 2019 and 2022 [24].

The observed surge is consistent with the hypothesis of an “immunity gap” or “immunity debt,” whereby reduced exposure to common pathogens during confinement created a larger pool of susceptible children, leading to more infections once circulation resumed. Comparable trends have been described in France, Germany, and other EU countries, particularly for RSV, although the magnitude and timing varied according to local NPIs and reopening strategies [15,25]. Portuguese data appear to mirror these patterns, showing a delayed but pronounced increase in mild ARIs rather than a surge in severe disease.

Respiratory pathogen transmission usually follows seasonal cycles linked to climate and collective behavior. In Portugal, circulation peaks between late autumn and early spring [25], as also observed in Pre-C. During the pandemic, containment measures disrupted this rhythm, suppressing winter peaks and giving rise to off-season outbreaks in summer and early autumn. After restrictions were relaxed in early 2022 [26], the seasonal pattern gradually re-emerged, with more cases recorded in autumn/winter. Similar shifts in RSV and other respiratory viruses have been documented worldwide, supporting the notion that NPIs temporarily reshaped viral ecology [15,27].

This study enhances understanding of the pandemic’s impact on pediatric respiratory infections in Portugal. Strengths include the long observation window and robust clinical characterization. However, several limitations should be acknowledged. Data were sampled from the first five days of each month and from a single hospital, which may limit national generalizability. Some medical records lacked clear diagnostic detail. The study window may not fully capture the long-term consequences of COVID-19, and post-pandemic parental behavior, possibly influenced by increased health literacy, could have affected ED utilization.

Overall, this research underscores how NPIs, while essential for pandemic control, also reshaped the epidemiology of common childhood infections. Initial concerns that children might develop immune deficits leading to more severe disease were not supported in this cohort; nevertheless, larger multicenter studies are needed to assess the broader pediatric impact at the national level.

Finally, some methodological aspects warrant consideration. The sampling strategy, analyzing only the first five days of each month, may introduce sampling bias and affect the accuracy of seasonality estimates. In addition, denominator effects could influence comparisons between periods. Reliability for pathogen-specific trends may be reduced by incomplete diagnostic coding and the fact that microbiological testing was performed selectively, based on clinical judgement. Finally, our analyses relied mainly on p-values, without reporting effect sizes or adjusting for multiple comparisons; future studies should incorporate these elements to provide a more nuanced assessment of the data.

## Conclusions

In recent years, uncertainty about the short- and long-term impact of COVID-19 on public health has been a cause for concern. This retrospective study provides insight into how the pandemic and related measures influenced respiratory infections in children in our region. NPIs, such as physical distancing, mask use, frequent hand hygiene, and adequate ventilation, played a pivotal role, not only in curbing the spread of SARS-CoV-2 but also in reducing transmission of other respiratory pathogens with similar routes. Combined with progressive population immunization, these measures likely contributed to lower infection.

An increase followed the easing of restrictions in ARI cases, yet no parallel rise in disease severity or hospitalization burden was observed. This suggests that the post-pandemic return to normal exposure did not lead to significant complications related to an immune deficit; instead, it may reflect an adaptive immune response that is expected to stabilize as exposure continues. Nevertheless, our ability to detect subtle changes in disease severity or longer-term outcomes was limited by the study design and follow-up period. Further multicenter research with extended observation is warranted to confirm these findings and to better define the enduring impact of COVID-19 and NPIs on pediatric respiratory health.
